# Natural History and Therapeutic Response in Ophthalmic Plasminogen Deficiency: Two Cases and Call for Registry-Based Data

**DOI:** 10.3390/ijms27209373

**Published:** 2026-10-21

**Authors:** Sayeed Khan, Ashtyn Vogt, Osman Khan, Jeremy Tan, Sanober Nusrat

**Affiliations:** 1 Department of Ophthalmology, Albany Medical College, Albany, NY 12208, USA; 2 Department of Ophthalmology, Dean McGee Eye Institute, Oklahoma City, OK 73104, USA; ashtyn.vogt@ouhealth.com (A.V.); jeremy.tan@ouhealth.com (J.T.); 3 The Jimmy Everest Section of Pediatric Hematology-Oncology, Department of Pediatrics, University of Oklahoma Health Sciences Center, Oklahoma City, OK 73104, USA; osman-khan@ou.edu; 4 Hematology-Oncology Section, Department of Internal Medicine, University of Oklahoma Health Sciences Center, Oklahoma City, OK 73104, USA

**Keywords:** plasminogen deficiency, type 1, ligneous conjunctivitis, plasminogen, conjunctival diseases, rare disease

## Abstract

Plasminogen deficiency is an ultra-rare disorder of fibrinolysis that most commonly presents as ligneous conjunctivitis. Ophthalmic manifestations often precede systemic diagnosis, making ophthalmologists essential to early detection and management. We report two contrasting cases illustrating the spectrum of disease and therapeutic response. A 65-year-old woman with untreated type 1 plasminogen deficiency developed recurrent ligneous conjunctivitis leading to enucleation and multisystem morbidity, while a 4-year-old girl with autosomal dominant type 1 disease achieved sustained remission following systemic plasminogen replacement therapy (Ryplazim). These cases highlight the evolution of management from supportive care to disease-modifying therapy and the critical importance of early recognition. Long-term outcomes and treatment durability remain poorly defined, underscoring the need for registry-based collaboration to better characterize disease progression, therapeutic response, and quality-of-life impact in plasminogen deficiency.

## 1. Introduction

Plasminogen deficiency (PD) is a rare congenital disorder of fibrinolysis that leads to fibrin accumulation on mucosal surfaces, producing chronic, wood-like (“ligneous”) inflammatory lesions across multiple organ systems. Fibrin is the principal structural protein in blood clots and a key component of the wound-healing cascade [1]. Plasminogen is normally converted to plasmin, a serine protease that degrades fibrin during fibrinolysis [2]; in PD, impaired conversion disrupts this process, leading to persistent fibrin deposition and the formation of pseudomembranous lesions on mucosal surfaces [3]. PD arises from mutations in the PLG gene, leading to improper encoding of plasminogen [4,5,6,7]. While PD is well established in the literature, with ligneous conjunctivitis as the most common and pathognomonic manifestation, other mucosal sites such as the gingiva, middle ear, respiratory tract, and female genital tract can also be affected [4,6,7,8,9,10]. PD can be life-threatening when fibrin-rich pseudomembranes affect vital structures, including the upper airway, which may cause respiratory compromise, and, rarely, the central nervous system, where congenital occlusive hydrocephalus from ventricular fibrin deposition has been reported [4,7,9]. These systemic manifestations extend beyond ocular morbidity and underscore the importance of early recognition and coordinated multidisciplinary management to prevent irreversible complications [8,11].

The PLG gene, located on chromosome 6q26, encodes a mature 791 amino acid glycoprotein composed of an N-terminal activation peptide, five kringle domains that mediate lysine-dependent binding to fibrin and cell-surface receptors, and a C-terminal serine protease domain. The conversion to active plasmin occurs via cleavage of the Arg561-Val562 bond. Disease-causing PLG variants are predominantly missense mutations affecting the kringle or protease domains, though nonsense, splice-site, and compound-heterozygous genotypes have also been identified [12,13]. Most symptomatic individuals are homozygous or compound heterozygous, consistent with autosomal recessive inheritance, though heterozygous carriers with intermediate plasminogen levels and, less commonly, autosomal dominant transmission with reduced penetrance have been reported, as in our second patient [4,7]. Murine models support a causal role for plasminogen loss in ligneous disease: plasminogen-deficient mice spontaneously develop ligneous lesions of the conjunctiva and other mucosal surfaces that resolve with plasminogen reconstitution, paralleling the human phenotype and providing mechanistic support for replacement therapy [14].

PD is broadly classified into two major forms: type 1 (hypoplasminogenemia), representing a quantitative deficiency with reduced plasminogen antigen and activity, and type 2 (dysplasminogenemia), a qualitative defect in which antigen levels are normal but enzymatic activity is diminished [7,10]. Type 1 disease is clinically significant and accounts for nearly all symptomatic cases, including patients presenting with ligneous conjunctivitis, whereas type 2 deficiency is often asymptomatic and typically discovered incidentally [4,8,10,15].

Ophthalmic involvement is often the initial clinical manifestation of PD, commonly presenting before systemic disease is recognized [4,8,10,11]. Ligneous conjunctivitis is defined by recurrent, wood-like pseudomembranous lesions of the tarsal conjunctiva that are notably resistant to corticosteroids, antibiotics, and surgical excision, with recurrence not uncommon after attempted removal [7,8,10]. Ophthalmologists are typically the first specialists to encounter such patients and often play a pivotal role in early diagnosis and facilitating multidisciplinary management [8,16]. Despite the diagnostic significance of ocular findings, the clinical spectrum and optimal management of ophthalmic PD remain incompletely characterized, and treatment approaches are highly variable given the rarity of the condition and limited high-quality data.

Fewer than 200 cases of type 1 plasminogen deficiency (PLGD-1) have been reported worldwide, with most described as isolated case reports or small series, resulting in significant limitations in understanding the disease’s natural history, genotype-phenotype correlations, and treatment outcomes [6,9]. Ophthalmic involvement occurs in approximately 80–90% of affected individuals, most commonly as ligneous conjunctivitis, which frequently represents the initial presentation of the disease [4]. Onset is most often recognized in infancy or early childhood, though diagnosis may be delayed well into adulthood when presentations are atypical, isolated to a single site, or misattributed to infectious or allergic conjunctivitis, as illustrated by the decades-long course in our first patient. Reported supportive measures have included topical and subconjunctival heparin, corticosteroids, cyclosporine, alpha-chymotrypsin, hyaluronidase, and fresh frozen plasma, alongside repeated surgical debridement; each has offered, at best, temporary symptomatic relief, and mechanical removal of pseudomembranes has been repeatedly shown to provoke recurrence rather than resolution, likely by inciting further local fibrin deposition in the absence of corrected systemic fibrinolytic capacity [7,10,16].

Reported cohorts show a disproportionately high rate of parental consanguinity, consistent with the autosomal recessive inheritance pattern typical of type 1 disease and likely contributing to geographic clustering of cases in regions where consanguineous marriage is more common [4,7]. Genotype-phenotype correlation remains imprecise: patients with similar plasminogen activity levels can display markedly different disease severity and organ involvement, suggesting that modifying factors beyond the PLG genotype itself, such as local tissue trauma, mucosal microtrauma from environmental exposure, or concurrent inflammatory triggers, influence where and when pseudomembranous lesions develop. This variability underscores that plasminogen activity alone cannot reliably predict clinical trajectory, reinforcing the need for close longitudinal follow-up regardless of measured enzyme levels.

The introduction of intravenous human plasminogen concentrate (plasminogen, human-tvmh [Ryplazim^®^]) has transformed management by providing a disease-modifying therapy that restores systemic fibrinolytic activity [5,6,11,17]. Early clinical trials and post-marketing data demonstrate that regular administration of intravenous plasminogen concentrate (typically 6.6 mg/kg every 2–4 days, with dosing individualized based on clinical response and plasminogen levels) prevents recurrence of both ocular and extraocular lesions, with most adverse events being mild and no anti-plasminogen antibodies detected [5,11,17,18]. However, long-term ophthalmic outcomes and optimal dosing strategies remain undefined, and ongoing studies and registries are expected to address these knowledge gaps.

Most published accounts of ophthalmic PD are isolated case reports or small case series, each capturing a single point along the disease course and offering limited insight into how the timing of diagnosis and treatment shapes long-term outcome. To address this gap, we describe two patients who occupy opposite ends of that continuum within the same disease: an adult whose plasminogen deficiency went unrecognized for decades, resulting in progressive multisystem fibrin deposition and eventual enucleation, and a child diagnosed in early life who achieved and has sustained remission of ligneous conjunctivitis with systemic plasminogen replacement begun soon after presentation. Juxtaposing these two trajectories, allows us to showcase concretely what is gained by early recognition and what is lost by delay, and how dosing can be individualized to trough plasminogen activity in practice. In doing so, we add to the existing literature a paired, practice-oriented illustration of the consequences of diagnostic timing that can inform how ophthalmologists and hematologists triage refractory pseudomembranous conjunctivitis, together with a call for systematic, registry-based data, such as through the international HISTORY registry, needed to generalize beyond individual cases like ours. Data collection and evaluation were HIPAA-compliant, and this report adheres to the tenets of the Declaration of Helsinki.

## 2. Case 1 Presentation

### 2.1. Patient Information

A 65-year-old woman presented with recurrent ligneous conjunctivitis since early adulthood, ultimately requiring enucleation of the left eye for pain from corneal perforation and recurrent pseudomembrane formation. She reported progressive hearing loss, lifelong constipation, and recurrent oral mucosal lesions. Past medical history included bilateral ovarian masses resected in the 1980s (benign) and an ischemic stroke in 2023. She denied respiratory or renal symptoms, and there was no family history of similar disease.

### 2.2. Clinical Findings

**Figure 1:**
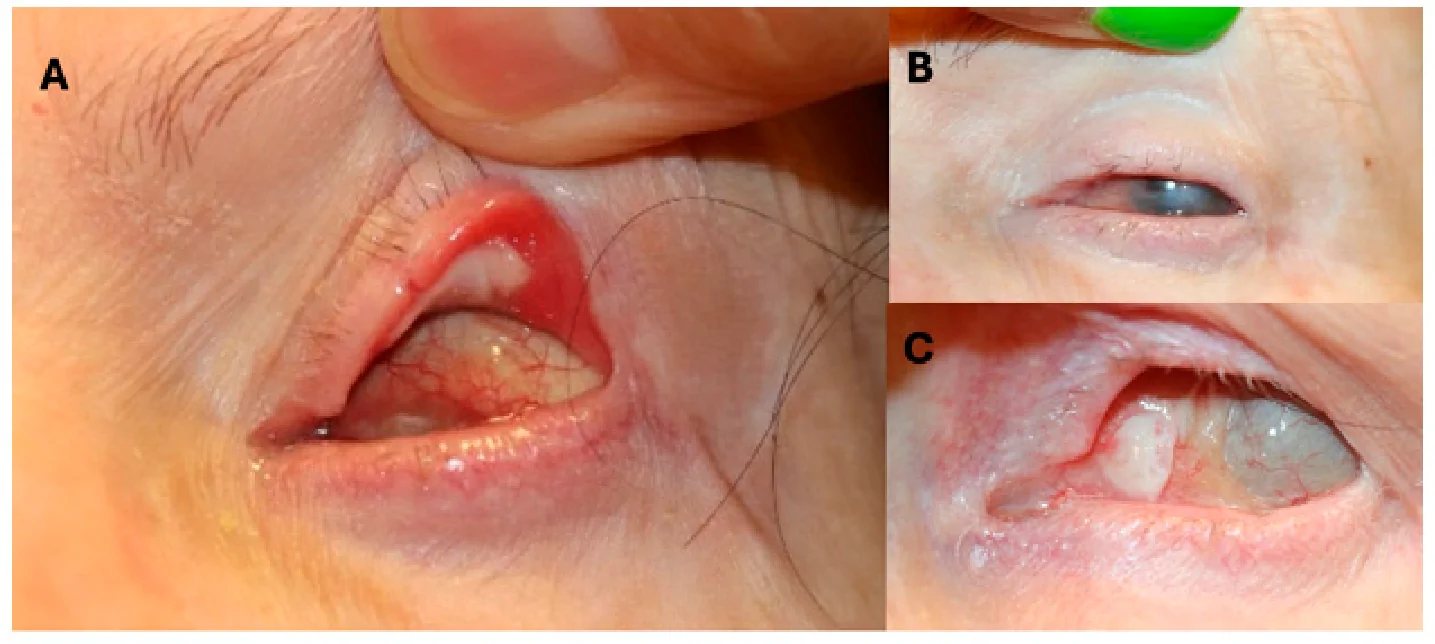
Ocular manifestations of longstanding ligneous conjunctivitis in type I plasminogen deficiency. (**A**) Recurrent plaque in the superior fornix. (**B**) Significant corneal breakdown over time leading to perforation, loss of vision, and pain. (**C**) Anophthalmic socket with new ligneous lesion forming.

Ocular disease was characterized by repeated episodes of thick, wood-like membranes involving the bulbar and tarsal conjunctiva (Figure 1A,B), unresponsive to corticosteroids, antibiotics, or repeated surgical removal. Progressive surface compromise led to corneal perforation and severe pain necessitating enucleation. The ocular surface remained quiescent for approximately one year afterward, until a new lesion developed in the medial fornix of the anophthalmic socket (Figure 1C). This occurred shortly before Ryplazim initiation and resolved rapidly after the first few doses, with no recurrence at 3 months. The right eye remained quiet at most recent examination; true clinical quiescence was achieved only after systemic therapy began.

### 2.3. Diagnostic Assessment

Laboratory evaluation revealed markedly reduced plasminogen levels: activity 29% (ref. 75–130%) and antigen 1.7 mg/dL (ref. 6–25 mg/dL), confirming type 1 hypoplasminogenemia. The constellation of ocular, otologic, gynecologic, and mucosal findings established multisystem PD, without evidence of hypercoagulability or systemic thrombosis.

### 2.4. Therapeutic Intervention

Given disease chronicity and low plasminogen levels, the patient was counseled on systemic replacement therapy: 6.6 mg/kg intravenously every 2–4 days (72 h apart initially), targeting trough activity roughly 10% above baseline. Potential adverse effects were reviewed, and a multidisciplinary care plan was established between hematology and ophthalmology, with infusions arranged under hematologic supervision and serial laboratory monitoring.

### 2.5. Follow-Up and Outcomes

At writing, systemic therapy had been initiated and the patient remained under close follow-up. Trough plasminogen activity increased by more than 20% above baseline, and dosing was changed to every 4 days. No new ocular or mucosal lesions were observed, and infusions were tolerated without adverse events; long-term monitoring is ongoing.

## 3. Case 2 Presentation

### 3.1. Patient Information

A 4-year-old girl with autosomal-dominant type 1 PD was evaluated for recurrent conjunctival pseudomembranes diagnosed as ligneous conjunctivitis. She was born full-term without perinatal complications, with no family history of ocular surface disease or abnormal bleeding.

### 3.2. Clinical Findings

At presentation in 2022, she exhibited thick, yellow white pseudomembranes of the tarsal conjunctiva with irritation and tearing. Lesions recurred rapidly after mechanical removal and were unresponsive to topical corticosteroids and antibiotics; no oral, nasal, or respiratory lesions were reported.

### 3.3. Diagnostic Assessment

Baseline plasminogen activity was 32–42% (ref. 75–130%) and antigen 5.2 mg/dL (ref. 6–25 mg/dL), confirming type 1 hypoplasminogenemia. Other hematologic parameters were normal, with no evidence of systemic fibrinolytic dysfunction or thrombosis.

### 3.4. Therapeutic Intervention

Systemic Ryplazim was initiated at 6.6 mg/kg intravenously every 3 days through an implanted port, guided by trough activity with a goal of 10–20 mg/dL above baseline. During an 11-month interruption in 2023–2024 due to national shortage, ocular irritation recurred but resolved promptly with resumption; trough activity increased 10–20% and dosing remained every 3 days. No new mucosal lesions developed, and there was no bleeding, dyspnea, or gastrointestinal involvement.

### 3.5. Follow-Up and Outcomes

On follow-up in 2024, the conjunctiva was clear without pseudomembranes. She remained off topical corticosteroids since July 2022, and systemic therapy was well tolerated aside from mild port-site tenderness, with no infections or hypersensitivity reactions. Continued infusions are planned twice weekly, with hematology and ophthalmologic monitoring every 3 months.

## 4. Discussion

### 4.1. Spectrum and Timing of Recognition

PD exhibits a broad clinical spectrum, with outcomes determined largely by the timing of diagnosis and treatment initiation. Our two patients illustrate the extremes of this continuum: the adult’s decades-long natural history culminating in enucleation and multisystem morbidity contrasts sharply with the child’s early recognition and durable remission on systemic replacement therapy [5,6,9]. This divergence underscores that delayed identification permits irreversible fibrin deposition and scarring, whereas early diagnosis allows disease modification before structural damage occurs [5,6].

Our aim in presenting these trajectories side by side is to argue for earlier systemic recognition of PD, since only a diagnosis made before irreversible fibrin deposition and multiorgan involvement occur allows disease-modifying therapy to prevent the systemic, and not merely the ocular, complications that characterized our first patient’s course.

### 4.2. Diagnostic Cues and the Ophthalmologist’s Role

Diagnosis of type 1 PD presenting as ligneous conjunctivitis relies on recognizing recurrent, wood-like pseudomembranous lesions of the tarsal conjunctiva that are refractory to corticosteroids, antibiotics, and surgical excision, often bilateral with delayed epithelial healing [8,19,20,21]. These lesions may accompany impaired wound healing or fibrin-rich pseudomembranes at other mucosal sites, such as the gingiva, middle ear, or respiratory tract; their presence, especially in children, should prompt suspicion for a systemic fibrinolytic defect. Recommended workup includes plasminogen antigen and activity measurement, with genetic testing for PLG mutations when available [6,8,21,22].

Clinicians unfamiliar with this rare disorder, as well as patients and families, can access current diagnostic criteria, treatment information, and patient-support resources through the Plasminogen Deficiency Foundation (https://www.plasminogendeficiency.org/, accessed on 11 October 2026) [23], particularly useful given the frequent diagnostic delay documented in cases such as our first patient’s.

Histopathologically, ligneous conjunctivitis shows dense, acellular fibrin deposits within pseudomembranes with minimal inflammatory infiltrate and disrupted epithelium [21,24]. Immunohistochemical staining of biopsy specimens can demonstrate absent or markedly reduced plasminogen and plasmin activity, supporting the diagnosis.

Ocular involvement is the most common and often earliest manifestation of PD, frequently preceding recognition of mucosal or visceral disease by months to years and serves as a sentinel sign of impaired fibrinolysis [4,8,9,10,22]. Ophthalmologists are often the first to encounter affected patients, positioning the eye as a diagnostic window into this underrecognized systemic disorder. Early recognition of ocular disease not only prevents vision loss but may also mitigate downstream complications involving the middle ear, respiratory, and female genital tract [4,8,9,22].

Resolving this differential matters for reasons beyond nosology: each additional cycle of empiric treatment for a mimicking condition is a cycle of delay before the systemic workup that could avert complications such as those that accumulated in our first patient. Recognition of ligneous conjunctivitis requires distinguishing it from other causes of pseudomembranous or membranous conjunctivitis [8]. Adenoviral conjunctivitis typically presents acutely with follicular reaction, preauricular lymphadenopathy, and self-limited resolution over one to two weeks, in contrast to the chronic, recurrent course of ligneous disease [25]. Vernal keratoconjunctivitis produces giant papillae and shield ulcers rather than dense, wood-like membranes, and characteristically improves with topical antihistamines and mast-cell stabilizers, agents that provide little benefit in plasminogen deficiency [26]. Stevens-Johnson syndrome and toxic epidermal necrolysis cause acute cicatrizing conjunctivitis in the setting of systemic mucocutaneous sloughing and a clear temporal association with a triggering medication or infection, distinguishing them from the indolent, recurrent presentation typical of PD [8]. Ocular cicatricial pemphigoid, now more commonly classified within the broader category of mucous membrane pemphigoid, similarly produces progressive conjunctival scarring but is distinguished by symblepharon formation and linear IgG and C3 deposition along the conjunctival basement membrane on direct immunofluorescence, in contrast to the dense, acellular fibrin deposition seen in ligneous conjunctivitis; unlike plasminogen deficiency, its treatment centers on systemic immunosuppression and is often notoriously difficult to control [27]. Chemical or thermal injury is distinguished by clear exposure history and a stable, non-recurrent clinical course after the acute injury resolves. In equivocal cases, conjunctival biopsy demonstrating dense, acellular fibrin deposition with minimal inflammatory infiltrate, combined with plasminogen activity and antigen testing, is diagnostic and should be pursued whenever pseudomembranous conjunctivitis proves atypical or treatment-refractory [8,21].

**Figure 2:**
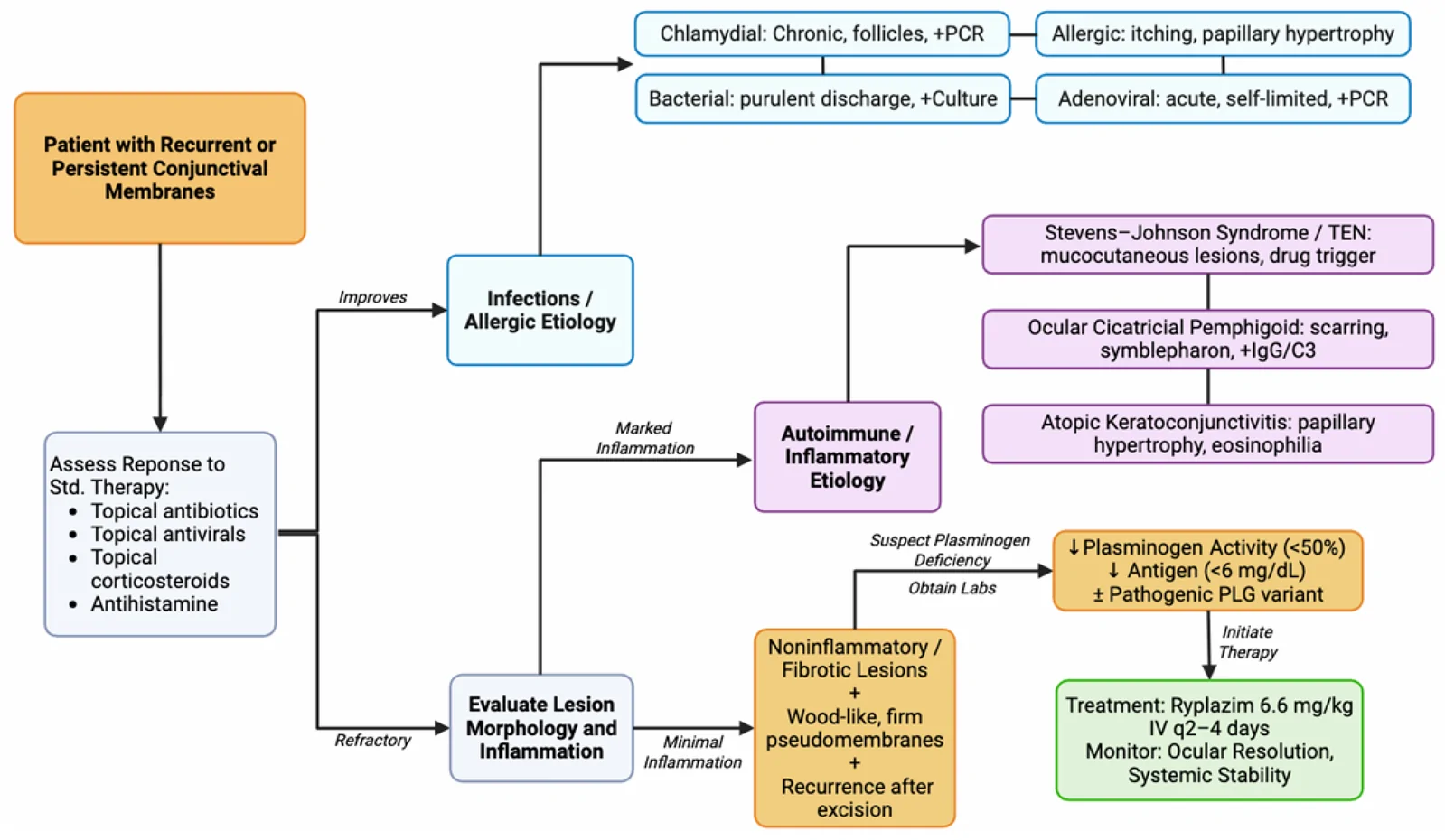
Diagnostic approach to recurrent pseudomembranous conjunctivitis and identification of plasminogen deficiency. Stepwise evaluation of patients presenting with recurrent or persistent conjunctival membranes. Initial assessment includes response to standard topical therapies to rule out infectious or allergic causes. Lack of improvement and the presence of recurrent, wood-like, noninflammatory pseudomembranes should prompt evaluation for plasminogen deficiency. Laboratory testing showing reduced plasminogen activity and antigen levels, with or without PLG gene mutation, confirms type I hypoplasminogenemia. Early systemic plasminogen replacement therapy (Ryplazim) can prevent recurrence and promote ocular and systemic remission.

Resolving this differential promptly, rather than cataloging it, is what let the stepwise pathway in Figure 2 translate into the systemic benefit demonstrated in our second patient: distinguishing PD from its ocular mimics is a means to earlier systemic management, not an end in itself.

Intravenous plasminogen concentrate acts by restoring circulating plasminogen to levels sufficient for endogenous conversion to plasmin at sites of fibrin deposition, thereby correcting the underlying enzymatic defect rather than simply treating its downstream manifestations. Population pharmacokinetic modeling has shown that plasminogen activity declines in a dose- and time-dependent manner following infusion, supporting individualized dosing intervals guided by trough activity rather than a fixed regimen for all patients [20]. This pharmacologic rationale is reflected in both of our cases, in which dosing frequency was titrated to trough plasminogen activity rather than administered on a fixed schedule, and it explains why our second patient’s disease recurred predictably once therapy was interrupted by drug shortage and resolved again upon reinstitution.

Figure 2 summarizes the diagnostic pathway we applied in both cases and that we propose more broadly for patients presenting with recurrent or treatment-refractory pseudomembranous conjunctivitis, particularly in settings where access to subspecialty hematology consultation may be delayed. Initial management should address common infectious and allergic etiologies with standard topical therapy; failure to respond, particularly when membranes recur rapidly after mechanical removal, should prompt consideration of plasminogen deficiency rather than repeated debridement alone. Plasminogen activity and antigen testing, supplemented by PLG genetic testing where available, establishes the diagnosis and distinguishes type 1 from type 2 disease. We propose that this stepwise approach, applied early rather than after prolonged, unsuccessful courses of conventional therapy, may shorten the diagnostic delay that characterized our first patient’s decades-long disease course. Because plasminogen activity and antigen testing are inexpensive, rapid, and widely available through standard coagulation laboratories, we suggest that these assays be obtained as soon as a patient is confirmed non-responsive to an initial antiviral and anti-inflammatory treatment trial, rather than deferred until after additional empiric courses or repeated debridement. Incorporating this threshold into routine ophthalmic practice, as reflected in Figure 2, could shorten the diagnostic interval, reduce unnecessary interventions such as repeated corticosteroid courses or surgical debridement, and lessen the duration of patient suffering illustrated by our first patient’s decades-long course.

Beyond the ocular findings themselves, the multisystem burden evident in our first patient, spanning otologic, gastrointestinal, and gynecologic involvement, illustrates that ophthalmic disease severity alone does not capture the full clinical impact of longstanding, untreated plasminogen deficiency. Coordinated management involving ophthalmology, hematology, and, where relevant, otolaryngology and gynecology is therefore warranted even when ocular disease appears to be the dominant or sole presenting feature, since subclinical involvement of other mucosal sites may otherwise go unrecognized until symptomatic.

This report has several limitations inherent to its design. As an observation of two patients, it cannot establish generalizable dosing thresholds or predict which patients are most likely to develop extraocular or systemic complications. Follow-up since initiation of systemic plasminogen replacement therapy remains relatively short in our first patient, and longer observation will be needed to confirm durability of the ocular response following decades of untreated disease. Laboratory monitoring in both cases relied on plasminogen activity and antigen assays performed at a single reference laboratory, and inter-laboratory variability in these assays has not been well characterized in the literature, which may affect the generalizability of the specific trough targets used here.

These limitations reinforce the value of multi-center, registry-based data collection, such as through the international HISTORY registry, to define optimal trough targets, characterize predictors of extraocular involvement, and establish standardized monitoring intervals across the diverse clinical presentations of type 1 plasminogen deficiency. The HISTORY registry itself illustrates both the need for, and the feasibility of, this approach: coordinated across 26 centers in 12 countries, it had enrolled 51 subjects, including 24 probands and 27 first-degree relatives, toward a target of up to 100 probands and their relatives, and it prospectively collects genotype-phenotype data, detailed treatment and dosing histories, standardized laboratory and global coagulation assay results, and a biorepository of DNA and plasma, the kind of systematic data that individual case reports such as ours cannot generate on their own. Comparable registries in other rare disorders show what sustained data collection of this kind can ultimately yield: the NHLBI Alpha-1-Antitrypsin Deficiency Registry, which followed 1129 patients across dozens of centers, was large enough to identify a subgroup, those with baseline FEV1 of 35–49% predicted, in whom augmentation therapy measurably slowed lung-function decline, a stratified benefit that no single-center case series could have established [28].

## Data Availability

The original contributions presented in the study are included in the article, further inquiries can be directed to the corresponding authors.
